# Research progress on the neural mechanism of light therapy improving cognitive function in depression: from retinal projections to neuroplasticity

**DOI:** 10.3389/fneur.2026.1768810

**Published:** 2026-07-07

**Authors:** Mengying Lin, Haihua Tian, Weiwei Xie, Tingting Wu, Ling Huang, Xiangping Wu, Yuanzhi Zhao

**Affiliations:** 1 Department of Psychiatry, Affiliated Kangning Hospital of Ningbo University, Ningbo, China; 2 Ningbo University, Ningbo, Zhejiang, China; 3 Department of Gynecology, Women and Children's Hospital of Ningbo University, Ningbo, China

**Keywords:** cognitive dysfunction, depression, light therapy, neural circuit, retinal projections

## Abstract

Cognitive impairment is a core and debilitating symptom of depression that severely impacts long-term patient prognosis and functional recovery. As a non-invasive physical intervention method, Light Therapy (LT) holds considerable therapeutic promise in improving the cognitive function of patients with depression. This review synthesizes clinical and preclinical evidence to delineate the complete mechanistic pathway of LT from the reception of peripheral light signals to the remodeling of central brain functions, that is, the regulatory pathway “from retinal projections to neuroplasticity.” We first summarize clinical efficacy and its parameter dependence. We then detail the retinofugal projections via intrinsically photosensitive retinal ganglion cells (ipRGCs) to key brain regions such as the suprachiasmatic nucleus (SCN), the limbic system (e.g., amygdala, raphe nucleus) and the thalamus, and their role as the “neural highway” for light signal transmission. Finally, we elucidate how these pathways converge to induce neuroplastic changes such as hippocampal neurogenesis, prefrontal synaptic remodeling, up-regulation of neurotrophic factors, neural oscillation entrainment and inhibition of neuroinflammation. By proposing an integrative “peripheral input-central processing-functional output” model, this review provides a comprehensive framework for understanding LT’s long-range brain regulation and a foundation for its precise clinical application.

## 1 Introduction

The Scientific Bridge Connecting “Light Input” and “Brain Changes” Globally, depression is one of the major mental disorders leading to disability, and its significant disease burden is largely driven by the persistent cognitive impairment throughout its course. This type of cognitive impairment involves multiple cognitive domains such as attention, executive function and working memory. Even after the clinical remission of depressive emotional symptoms, it may persist for a long time, becoming a core bottleneck restricting the recovery of patients’ social functions and the improvement of quality of life (1–3). From the perspective of the relationship between pathological mechanisms and clinical prognosis, existing studies have clearly confirmed that the severity of cognitive impairment is significantly positively correlated with the overall functional impairment level of patients with depression (4). Further studies have revealed that there is not a one-way influencing relationship between depressive symptoms and cognitive function, but a dynamic interaction. Moreover, the baseline cognitive level of patients can serve as an important predictor of the developmental trajectory of subsequent depressive symptoms (3). More notably, the cognitive impairment associated with depression may also significantly increase the risk of long-term neurodegenerative diseases (such as Alzheimer’s disease) in individuals. This finding further highlights the clinical importance of the long-term impact of such cognitive problems (5–7). Therefore, the development of intervention strategies that can effectively improve the cognitive function of depression has become a key research frontier in the field of psychiatry.

Among various intervention methods, LT shows unique advantages due to its non-invasiveness, high safety and relatively fast onset of effect. Its application scope has been successfully expanded from the classic seasonal affective disorder (SAD) to non-seasonal depression (8, 9). Compared with antidepressant drugs, LT has significant advantages such as lower cost, lower incidence of side effects and faster onset of effect, providing a safer option for the treatment of depression (10). In recent years, the technical forms of LT have been continuously expanded, covering multiple technical paths such as visible light stimulation and transcranial photobiomodulation (tPBM). As an emerging light intervention method, tPBM acts directly on brain tissue through near-infrared light to regulate neural activity. It has shown application potential in improving depressive symptoms in a number of preclinical and clinical studies, and its potential mechanism is believed to be related to enhancing the local metabolic level of the brain and optimizing the efficiency of oxygen utilization (11, 12). Although current clinical trials have confirmed that tPBM has good patient tolerance and preliminary efficacy, the development of its optimal treatment parameters (such as light intensity, duration of action, stimulation target) and standardized intervention plans still requires further high-quality randomized controlled trials (13, 14). While clinical applications advance, a critical gap remains in understanding the integrated neural circuitry that translates peripheral light exposure into sustained cognitive benefits.

**Figure 1:**
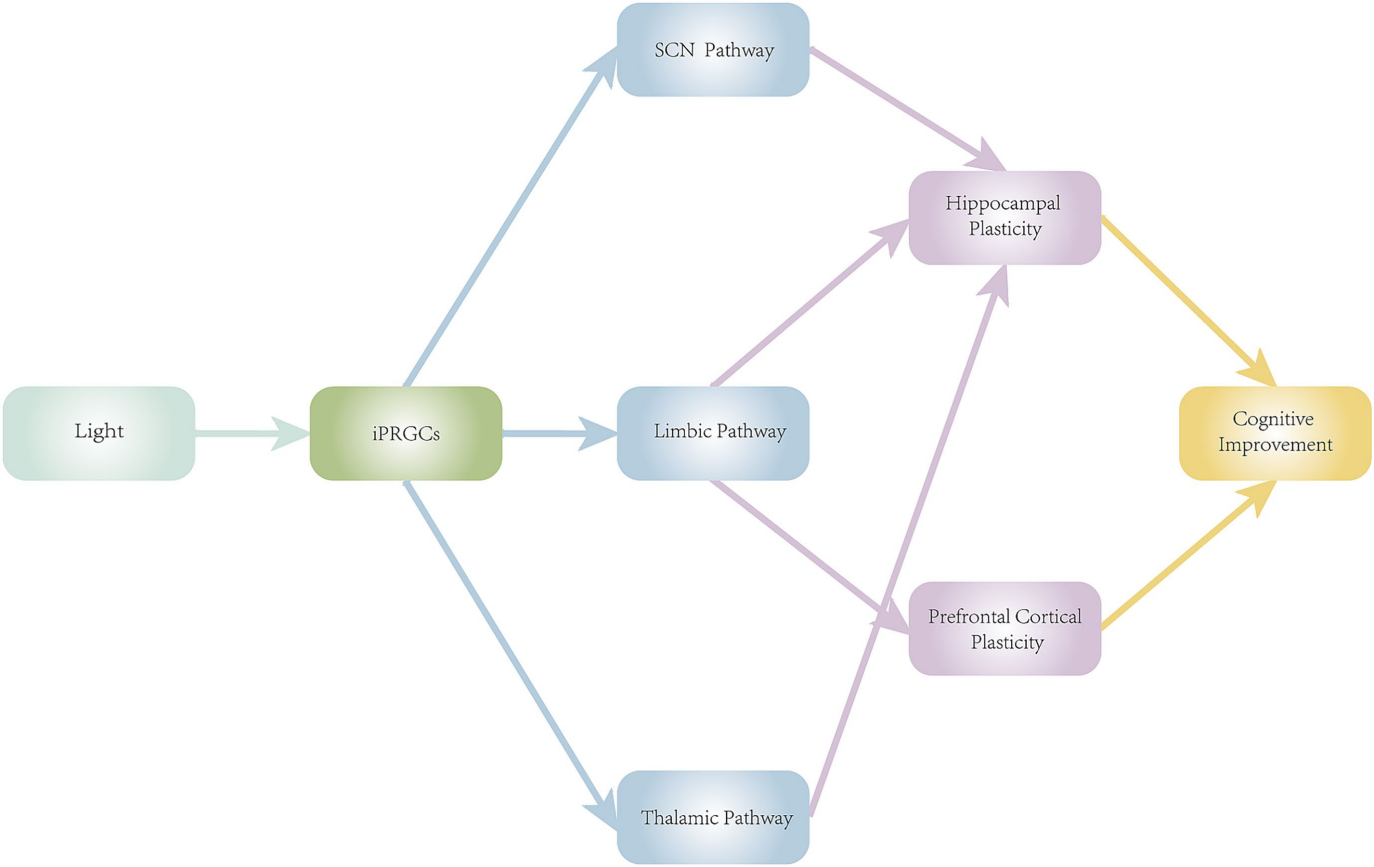
Schematic illustration of the proposed mechanistic framework through which light therapy improves cognitive function in depression. The pathway initiates with light input of specific parameters (1). This signal is received and integrated by intrinsically photosensitive retinal ganglion cells (ipRGCs) (2). IpRGCs then project these signals to downstream brain regions via distinct neural pathways (3). Including the retinohypothalamic tract (RHT) to the suprachiasmatic nucleus (SCN) for circadian entrainment, and projections to the amygdala, raphe nucleus, and thalamus. The activation of these pathways converges to induce neuroplasticity in key cognitive brain regions like the hippocampus and prefrontal cortex (4). Neuroplasticity Effects: After the activation of the above-mentioned pathways, a series of neuroplastic changes are eventually induced in key cognitive brain regions (mainly the hippocampus and prefrontal cortex), including neurogenesis, enhanced synaptic plasticity (increased dendritic spine density, long-term potentiation), up-regulation of neurotrophic factors such as brain-derived neurotrophic factor (BDNF), inhibition of neuroinflammation and optimization of neural oscillations (e.g., gamma oscillations) (5). Behavioral Output: The final effect is reflected in the improvement of depression-related cognitive functions (attention, memory, executive function).

The traditional accounts of LT’s mechanism, often limited to “circadian rhythm regulation” or “neurotransmitter modulation,” fail to delineate the complete causal chain from retinal signal reception to improved higher-order cognition. The title of this review, “From Retinal Projections to Neuroplasticity,” accurately summarizes this core scientific issue that needs to be clarified. Specifically, it is necessary to systematically answer two key questions: First, how do light signals be transmitted to the deep structures of the brain through specific neural projection pathways originating from the retina? Second, how do the signals from these long-distance projections eventually be transformed into long-lasting structural and functional changes (i.e., neuroplasticity) in the brain that support the improvement of cognitive function? To intuitively show this overall framework, this study constructs the following schematic diagram (Figure 1).

Centering on this main line, this article will systematically integrate clinical and basic research evidence, and elaborate in detail how LT finally achieves the therapeutic endpoint of neuroplasticity through the “bridge” of retinal projection pathways.

### 1.1 Literature search strategy

This narrative review synthesizes preclinical and clinical evidence on the mechanisms by which LT improves cognitive function in depression. Relevant English-language articles were identified through searches of PubMed, Web of Science, and Scopus up to July 2025. Search terms included combinations of “light therapy,” “photobiomodulation,” “bright light,” “depression,” “cognitive dysfunction,” “retinal projections,” “suprachiasmatic nucleus,” “amygdala,” “raphe,” “thalamus,” “neuroplasticity,” “BDNF,” “neurogenesis,” and “gamma oscillations.” Additional studies were retrieved from reference lists of key articles. Inclusion criteria encompassed mechanistic studies in animal models of depression or light manipulation, clinical trials/imaging studies examining LT’s cognitive effects in depressive disorders, and reviews that provided mechanistic insights. For Table 1, we selected representative clinical studies that specifically assessed cognitive outcomes or related brain changes following LT. Studies solely investigating circadian rhythm without cognitive endpoints were excluded. The synthesis aimed to construct an integrative model rather than to exhaustively survey all published literature; therefore, the review is best described as a narrative, hypothesis-driven synthesis.

**Table 1:** Summary of selected clinical studies on light therapy for depression.

Source	Intervention	Control	Time	Diagnosis	Results	Key limitations
Strong et al. (88)	470 nm blue light 45 min (6:00 and 8:00 a.m.)	650 nm red light 45 min (6:00–8:00 a.m.)	3 weeks	SAD	That light therapy using nar-row-band LED panels emitting wavelengths of 470 nm is effective in the treatment of SAD	Small sample size; limited generalizability
Chen et al. (27)	10,000 lux 30 min (8:00–17:00) 200 lux 30 min (8:00–17:00)	None	14 days	Sub-threshold depression	The bright light therapy (BLT) group showed significantly higher remission and effectiveness rates	Short treatment duration; lack of long-term follow-up
Liu et al. (89)	Blue light with a specific wavelength 30 min	None	8 weeks	Post-stroke depression	Phototherapy can improve depression, increasing the levels of BH4 and Trp in plasma while decreasing the level of BH2, thereby reducing the levels of TNF-*α*, IL-6, and IL-1β in plasma	Open-label design; no sham control
Chojnacka et al. (90)	10,000 lux 30 min (8:00–9:00)	None	14 days	Non-seasonal de-pression	The depression scores of the BLT group did not show overall improvement compared to the control group, but its remission rate and response rate were both significantly higher	No placebo control; short intervention period
Güzel Özdemir et al. (91)	7,000 lux 60 min	None	4 weeks	MDD	BLT induced significantly stronger and more rapid beneficial effects the in depressive mood of patients	Relatively small sample size; single-center design
Noda et al. (92)	Violet light parameters (375 nm, 40 Hz, 310 μW/cm^2^)	Violet light parameters (375 nm, 40 Hz, 10 μW/cm^2^)	4 weeks	Mild MDD	Exposure to 40 Hz violet light can improve mild depression	Pilot study with limited sample; short follow-up
Zhou et al. (18)	5,000 lux 60 min (6:30–9:00)	<100 lux 60 min (6:30–9:00)	17 days	BD (de-pressed phase)	BLT showed a greater ameliorative effect on bipolar depression than the control	Short treatment period; adjunctive therapy limits attribution
Chen et al. (19).	5,000 lux 30 min	<5 lux 30 min	8 weeks	Sub-threshold depression	BLT improved depressive symptoms and attention/vigilance	Small sample; single-blind design

## 2 Clinical efficacy and optimization: behavioral endpoints of neuroplasticity

Clinical efficacy, as an intuitive external manifestation of underlying neural mechanisms, serves as the core evidence for verifying that LT improves cognitive function in depression. A large number of studies have shown that LT can not only effectively reduce the core symptoms of depression (15–17), but also directly improve the multi-dimensional cognitive function of patients. In the population of patients with non-seasonal depression, especially the elderly subgroup, LT can significantly reduce the severity of depressive symptoms (15, 16); in patients with bipolar depression, LT has also been confirmed to reduce the severity of the disease, showing good clinical application potential (17, 18). For example, a randomized controlled trial on the population with subclinical depression showed that after 8 weeks of LT intervention, the performance of patients in attention and alertness tests was significantly improved (19). More importantly, the study suggests that such cognitive improvement may be related to the functional reorganization of brain networks, such as regulating the functional connection between the cerebellum and the default mode network and this process itself is a typical manifestation of system-level neuroplasticity.

Although the existing evidence generally supports the positive impact of LT on cognitive function, its efficacy has clear cognitive domain specificity and individual heterogeneity. Studies have shown that the improvement effect of LT on attention and working memory is relatively clear, while its impact on some advanced executive functions (such as decision-making ability) has not yet formed a consistent conclusion (20–22). The heterogeneity of the cognitive improvement effect of LT precisely suggests that there is a complex neural circuit and plasticity regulation mechanism behind it, and this effect is affected by a variety of factors:

- Circadian clock type: Morning-type individuals usually have a better response to LT, suggesting that their inherent rhythm system (SCN pathway) is more likely to be synchronized by light cues (23);
- Disease subtype: Whether there are comorbidities such as anxiety (24) baseline severity of depression (25) (e.g., patients with dementia combined with severe depression have more significant LT efficacy), etc., will affect the treatment effect. This may be related to the differences in the excitability of the retina-limbic system pathway (e.g., amygdala) among different patient groups;
- Treatment parameters: The effect of LT shows significant parameter dependence. Different treatment parameters may preferentially activate different retinal projection pathways, thereby triggering differentiated neuroplastic effects. The effect of LT is highly dependent on parameter settings, including wavelength, intensity/dose and timing. These parameters jointly determine the characteristics of the visual and non-visual effects of the treatment. As summarized in Table 1, clinical studies have employed diverse parameters, including wavelengths from 375 nm to 470 nm, intensities from 5,000 to 10,000 lux, and exposure durations of 30–60 min. Wavelength: Blue light (460–480 nm) optimally activates ipRGCs (26); longer wavelengths (red/NIR) are used for tPBM due to deeper tissue penetration and mitochondrial effects (11, 12). Intensity: sufficient illuminance is required to engage non-visual pathways, while excessively high intensities may cause discomfort or glare (16, 27–30). Timing: Morning light advances the circadian phase, while evening light delays it (31, 32); matching timing to individual chronotype is critical for maximizing efficacy.

Limitations and Critical Evaluation: Although existing evidence generally supports a positive effect of LT on certain cognitive domains, the literature is not uniformly positive. Several studies have reported null or inconsistent effects on higher–order executive functions (20–22). Moreover, many of the available clinical trials are characterized by relatively small sample sizes and inherent difficulties in adequate blinding, which may influence the robustness of the conclusions. These factors underscore the need for larger, well-controlled studies.

Safety Considerations: Light therapy is generally well tolerated, with the most commonly reported side effects being headache and eyestrain (17, 18). In patients with bipolar depression, there is a potential risk of switching into hypomania or mania; therefore, LT should be used with caution and ideally combined with a mood stabilizer (17, 18). Ocular safety is also an important consideration, particularly with blue-enriched light, as prolonged exposure may damage retinal ganglion cells (30).

We have summarized the application of light therapy in depression in Table 1, which now includes a“Key Limitations” column to critically synthesize the evidence.

The above results suggest that the heterogeneity and parameter sensitivity of the clinical efficacy of LT may be due to the differential regulatory effects of different parameter combinations on downstream neuroplastic events. Therefore, in-depth understanding of the functional characteristics of the following retinal projection pathways is a necessary prerequisite for the realization of the precise application of LT.

## 3 Central pathway I: retinofugal projections—the neural highways for light signal transmission

**Figure 2:**
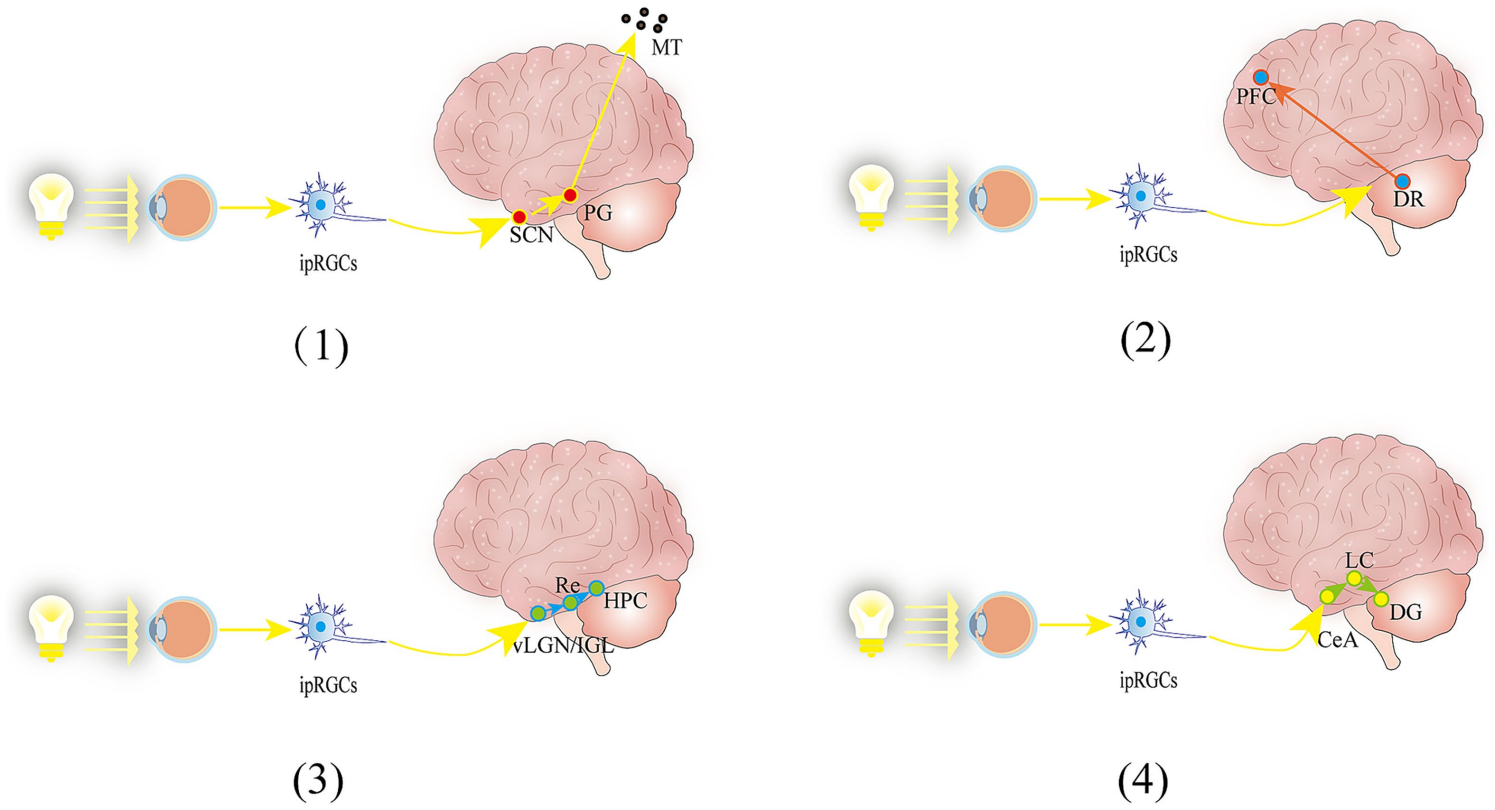
Core retinofugal projection pathways mediating the non-image-forming effects of light therapy. The schematic sagittal view of a mouse brain highlights four major pathways originating from ipRGCs: (1) Retina → SCN → Pineal Gland pathway (yellow arrow) for circadian rhythm resetting; (2) Retina → Amygdala (CeA) → Locus Coeruleus (LC) → Hippocampal Dentate Gyrus (DG) pathway for rapid emotional and memory regulation; (3) Retina → Dorsal Raphe Nucleus (DR) → Prefrontal Cortex (PFC) pathway for serotonergic system modulation; (4) Retina→Ventrolateral Geniculate Nucleus/Intergeniculate Leaflet (vLGN/IGL) → Thalamic Reuniens Nucleus (Re) → Hippocampus (HPC) pathway for direct spatial memory regulation.

The regulation of the brain by light signals starts from the retina, among which ipRGCs are the only “starting point” and “signal distribution station” in this process (33). ipRGCs can not only directly sense light through the melanopsin they express, but also integrate visual input signals from traditional rod cells and cone cells, and construct a complex projection network specifically serving non-image-forming visual functions through their axons. This network can accurately transmit environmental light information to multiple subcortical brain regions directly related to emotion and cognition (34, 35). The neuroplastic effect mediated by the retina-brain pathway is of key scientific significance for revealing the mechanism of action of LT (36). These projection pathways are the structural basis for understanding the “long-distance” brain regulation of LT. To clearly show this complex network, this study draws the following schematic diagram (Figure 2).

### 3.1 Rhythm resetting pathway: retina-SCN projection and its downstream cascade effects

In the context of LT, the retina-SCN pathway serves as a primary route through which light signals reset the central circadian pacemaker, thereby creating optimal physiological conditions for neuroplasticity and cognitive enhancement. The retina-SCN pathway is the most in-depth studied LT-related projection pathway so far, and its specific path is: ipRGCs → Retinohypothalamic Tract (RHT) → SCN (34). As the core circadian pacemaker of the body, the normalization of SCN function is a prerequisite for the realization of long-term and stable neuroplasticity, and plays a key role in integrating light signals, maintaining the homeostasis of endogenous rhythm and regulating cognitive function (37–39). Clinical studies have confirmed that the abnormal function of SCN in adolescents is closely related to the occurrence of depression associated with circadian rhythm disorders, suggesting that the functional state of SCN may be an important intervention target for depression-related cognitive impairment (40).

The SCN does not directly process advanced cognitive information, but creates macro-physiological conditions for neuroplasticity by regulating downstream effectors, which specifically include:

Pineal Gland-Melatonin Axis: The neural signals of the SCN finally inhibit the synthesis of melatonin in the pineal gland during the day through multi-level relays. LT regulates the rhythm of melatonin secretion through this axis, thereby stabilizing the sleep-wake cycle. Sufficient sleep (especially slow-wave sleep and rapid eye movement sleep) is believed to be crucial for hippocampal memory consolidation and synaptic homeostasis regulation. Accordingly, it has been proposed that this pathway may indirectly support cognitive-related neuroplasticity by optimizing sleep, a key state for brain self-repair and remodeling (41). Animal studies have further confirmed that exogenous supplementation of melatonin can improve depression-like behaviors and restore the level of 5-HT in the brain (42).

Hypothalamic-Pituitary-Adrenal (HPA) Axis and Glucocorticoid Rhythm: The SCN also regulates the activity of the HPA axis through a complex neural network, making plasma glucocorticoids (such as cortisol) show a clear circadian rhythm. Glucocorticoid receptor expression in the hippocampus is high, and the hippocampus itself can act as a peripheral oscillator; the SCN ensures the normal oscillation of local clock genes (such as PER1) in the hippocampus by maintaining the stability of the glucocorticoid rhythm. Studies have shown that the rhythmic expression of hippocampal PER1 is a necessary condition for the “daytime-dependent” regulation of synaptic plasticity and learning and memory processes (43, 44). Therefore, this pathway is thought to contribute to the learning and memory process by ensuring that the rhythmic time window for hippocampal plasticity remains optimally phased.

The ventrolateral core region of the SCN is the key part for light signal integration, as the vasoactive intestinal peptide (VIP) neurons enriched in this region can directly receive RHT projections. VIP neurons maintain the highly synchronized electrical activity of SCN neurons through spontaneous Ca^2+^ activity, light-induced Ca^2+^elevation, and the VIP-VPAC2 signaling pathway, thereby ensuring circadian homeostasis (45). Beyond rhythm regulation, VIP neurons also mediate light-induced transient amnesia via the SCN → paraventricular nucleus of the thalamus (PVT) circuit. Acute exposure to strong light activates these neurons, selectively impairing the retrieval of hippocampus-dependent trace fear memory (which recovers within 24 h). Artificial modulation of VIP neuron activity can replicate or block this effect, broadening the role of the SCN in memory regulation (46).

The effects of different light conditions on SCN function and cognition are bidirectional. Appropriate light (e.g., 480 nm blue light) enhances the amplitude of SCN circadian rhythms, stabilizes neural stem cell gene expression, and alleviates cognitive decline induced by sleep deprivation (47). In human studies, higher light intensity reduces abnormal hypothalamic activity and improves executive function, with cognitive performance negatively correlated with SCN neuron activity. This suggests that light may optimize rhythm synchronization to enhance cognition (48). Conversely, aberrant light exposure at night disrupts SCN rhythm stability and worsens cognitive deficits. The enhanced activity of the ventral SCN under appropriate light positively correlates with cognitive improvement, further confirming the SCN’s central role in light-mediated cognitive regulation (47, 48).

### 3.2 Rapid regulation pathway: direct projections from the retina to the limbic system and thalamus

In recent years, breakthrough studies have revealed that ipRGCs give rise to numerous pathways that bypass the SCN and directly project to core emotion-and cognition-related brain regions (35, 49, 50).

#### 3.2.1 Retina-amygdala projection and emotion-cognition integration

This projection enables environmental light to directly and quickly affect the activity of the amygdala.

A mouse study identified a micro-mechanism whereby moderate white light (400 lux) activates specific corticotropin-releasing factor (CRF)-positive neurons in the central amygdala (CeA) to initiate a continuous projection loop of “CeA → Locus Coeruleus (LC) → Hippocampal Dentate Gyrus (DG)”; this loop finally enhances the spatial memory retrieval ability directly in the DG region by releasing NE and acting on *β*-adrenergic receptors (51). This finding clearly reveals a complete “retina → behavior” regulatory pathway, in which the amygdala acts as a key relay station to directly convert light signals into regulatory signals for hippocampal plasticity.

In human studies, functional magnetic resonance imaging (fMRI) shows that bright light can inhibit the activity of the amygdala in the resting state and enhance its functional connection with the dorsolateral prefrontal cortex (dlPFC) (52, 53). A randomized controlled trial on young people with subthreshold depression further confirmed that blue light therapy (BLT) can specifically regulate the functional connection between the amygdala subregions and brain regions such as the thalamus and middle temporal gyrus. These connection changes are closely related to the serotonergic system and can predict the antidepressant efficacy of BLT (54). These findings directly link the retina-amygdala projection with the improvement of human emotions and potential brain network plasticity.

In addition to direct projections, the pathway interaction between the amygdala and the hippocampus also provides supplementary evidence for cognitive regulation: studies have found that the amygdala has a one-way dominant regulation on the hippocampus, which is manifested in that the amygdala *θ*/*α* oscillation directly regulates the hippocampal *γ* oscillation; this cross-brain region oscillation coupling is the core mechanism of cognitive processes such as memory encoding and retrieval, suggesting that the retina-amygdala pathway may be indirectly involved in cognitive regulation by regulating the amygdala-hippocampus oscillation coupling (55).

#### 3.2.2 Retina-raphe nucleus projection and monoaminergic regulation

ipRGCs can directly project to the brainstem raphe nucleus (especially the dorsal raphe nucleus, DR), which is the main origin of serotonergic neurons in the brain (56, 57). In terms of signal transmission mechanism, retinal ganglion cells transmit light signals to the raphe nucleus through axon collaterals, thereby regulating the activity of 5-HT neurons. Studies have confirmed that this pathway can be involved in regulating the defensive response of animals to rapidly approaching visual threats by inhibiting 5-HT neurons in the raphe nucleus, providing experimental evidence for its physiological function (58). Further studies have revealed the specific role of different functional types of retinal ganglion cells: ON-type cells mainly activate GABAergic neurons in the raphe nucleus, while OFF-type cells tend to activate 5-HT neurons (57). This differentiated projection mechanism indicates that retinal input can precisely modulate the neural activity of the raphe nucleus, thereby affecting emotional and cognitive functions.

Experiments on light stimulation of the dorsal raphe nucleus show that this operation can not only enhance the activity of local neurons, but also observe hemodynamic changes in the whole brain through fMRI, suggesting that the release of 5-HT induced by light stimulation can activate widely distributed 5-HT receptors, which may be involved in emotional and cognitive regulation (59). In addition, the raphe nucleus can also affect hippocampal function through its 5-HTergic and glutamatergic neurons, providing a direction for exploring the downstream pathway of LT in improving the cognitive function of depression (60). As the main source of 5-HTergic neurons, the dense projection of the raphe nucleus to the medial prefrontal lobe further amplifies the regulatory effect of light on PFC function, forming a “light-raphe nucleus-prefrontal lobe” regulatory axis (61).

Light stimulation regulates the excitability of 5-HTergic neurons in the raphe nucleus through this projection, thereby regulating the level of 5-HT release in its projection target areas (such as the prefrontal cortex, hippocampus, amygdala). Therefore, the retina-raphe nucleus projection is well-positioned to contribute to the neurochemical environment that supports positive plastic changes in these brain regions by providing appropriate 5-HTergic tone for the prefrontal cortex and hippocampus (49, 62).

#### 3.2.3 Retina-thalamus projection and direct cognitive regulation

The thalamus is not only a relay station for visual information, but also its specific nuclei receive direct projections from ipRGCs, forming an important pathway for regulating cognition and emotion, which specifically includes:

Retina-vLGN/IGL-Reuniens Nucleus (Re) Pathway: Studies have found that long-term exposure to strong light can specifically and reversibly enhance the spatial memory ability of mice through the two-synapse pathway of “retina → Ventrolateral Geniculate Nucleus/Intergeniculate Leaflet of the Thalamus (vLGN/IGL) → Reuniens Nucleus of the Thalamus (Re).” Mechanistically, specific ON-type ganglion cells in the retina activate CaMKIIα-positive neurons in the vLGN/IGL, which then activate similar neurons in the Re nucleus; more importantly, the Re nucleus has been confirmed to be a common key node for light to improve memory and enhance hippocampal gamma (*γ*) oscillations (35). This finding is the first to clarify a specific visual circuit that starts from the retina, relays through the thalamus, and directly regulates hippocampal rhythm and memory function.

Emotion-related Thalamic Pathway: The projection of ipRGCs to the perihabenular nucleus (PHb) of the thalamus has been confirmed to be a “necessary and sufficient” pathway for light to regulate emotional behaviors (50); while the retina-vLGN-Lateral Habenula (LHb) pathway has been confirmed to be involved in alleviating stress-induced depression-like behaviors, and its emotional improvement effect can indirectly promote the recovery of cognitive function (63).

Recent studies have further clarified that the independent activation of ipRGCs (without relying on blue light perception or cone cell participation) can regulate the processing of cognitive tasks, indicating that it can act as an independent functional unit to directly participate in the cognitive regulation network, providing a new target for the development of LT strategies targeting ipRGCs (26).

## 4 Central pathway II: convergence on neuroplasticity—the final common path for cognitive enhancement

### 4.1 Hippocampal plasticity: neurogenesis, synaptic strengthening and microenvironment improvement

LT exerts profound effects on hippocampal plasticity, a key substrate for learning and memory, through multiple converging mechanisms. The hippocampus is a key center for learning and memory, and also one of the brain regions with the most significant neuroplasticity. The regulation of hippocampal plasticity by LT is mainly reflected in the following three aspects:

#### 4.1.1 Promotion of neurogenesis

In the adult brain, the hippocampal dentate gyrus (DG) is one of the few brain regions that can continuously produce new neurons, a process known as adult hippocampal neurogenesis. Studies have found that long-term exposure to bright light can effectively promote the neurogenesis process in the DG region of the hippocampus of adult rats. In rodent models, long-term exposure to bright light has been shown to effectively promote the neurogenesis process in the DG region of the hippocampus of adult rats. The newly proliferated neural precursor cells can further differentiate into mature neurons and be successfully integrated into the existing hippocampal neural circuit; moreover, the cognitive improvement at the behavioral level (such as the improvement of performance in the Y-maze and novel object recognition tests) is significantly positively correlated with the degree of neurogenesis (64). At the clinical level, a 4-week randomized controlled study observed through magnetic resonance imaging (MRI) that the volume of the left hippocampal DG of patients receiving blue light therapy increased significantly. This result indirectly suggests that LT may also induce neurogenesis or structural hypertrophy in the human brain (65). BDNF is widely regarded as a has been shown to candidate molecule mediating this process: experimental BDNF infusion into the hippocampus promotes neurogenesis (66), supporting its role as a key mediator. Furthermore, its expression level is directly related to the formation of long-term memory-the lack of PER1 can lead to impaired long-term memory, while light can optimize the memory formation process by targeting the regulation of hippocampal PER1 expression and BDNF level (67).

#### 4.1.2 Enhancement of synaptic plasticity

Synaptic plasticity refers to the ability of synaptic connection strength to change with activity experience, among which long-term potentiation (LTP) is considered as a cellular model of learning and memory. Evidence from animal studies suggests that light can enhance the synaptic efficiency of the hippocampus. The proposed molecular mechanism involves that light induces the influx of Ca^2+^ in the hippocampal region, activating the phosphorylation of calcium/calmodulin-dependent protein kinase II (CaMKII) and cAMP response element-binding protein (CREB); phosphorylated CREB (pCREB) enters the nucleus to initiate the transcription of genes such as BDNF; after the secretion of BDNF protein, it binds to the high-affinity receptor tyrosine kinase B (TrkB), activating downstream signaling pathways such as MAPK/ERK and PI3K/Akt, and finally leading to the increased expression of synaptic-related proteins such as synaptophysin and postsynaptic density protein 95 (PSD-95), driving the structural remodeling and functional strengthening of dendritic spines (68, 69). In addition, light can also regulate the rhythmic expression of synaptic proteins and the activity of neurotransmitters, participating in the dynamic regulation of cognition (70).

#### 4.1.3 Improvement of the neural microenvironment

Chronic stress and depression are often accompanied by increased levels of hippocampal neuroinflammation and oxidative stress, which can inhibit neurogenesis and synaptic plasticity. Preclinical studies indicate that LT and transcranial photobiomodulation can reduce astrogliosis and microglial activation in the hippocampus, accompanied by decreased levels of pro-inflammatory cytokines such as TNF-*α* and IL-6 (71). Moreover, appropriate light exposure can counteract the increase in hippocampal oxidative stress induced by insufficient light, and photobiomodulation can reverse this process (72). Therefore, LT creates a favorable microenvironment for hippocampal neurogenesis and synaptic plasticity through anti-inflammatory and anti-oxidative stress effects.

It should be noted that the regulation of hippocampal cognitive function by light has parameter-dependent effects, and wavelength, intensity and duration of action will all affect the final effect, which provides a basis for the optimization of clinical protocols. For example, blue light exposure may cause the down-regulation of hippocampal synaptic plasticity-related proteins, suggesting that different wavelengths may regulate cognition through different mechanisms, and parameters need to be accurately selected according to the treatment goal (73). In addition, the regulation of hippocampal rhythm and cognition by light is not limited to PER1, but also has a significant impact on the expression oscillation of PER2 and CRY2: abnormal light-dark cycles can lead to the rhythm disorder of hippocampal PER2 and CRY2, accompanied by the dysregulation of the expression of neuroplasticity-related genes (such as Gria1/2) and neurotransmitter receptor genes (such as Drd1), suggesting that the PER/CRY family may jointly participate in the light-mediated cognitive improvement by synergistically maintaining the hippocampal molecular homeostasis (74).

### 4.2 Prefrontal cortex plasticity: structural remodeling, molecular regulation and network optimization

Beyond the hippocampus, LT also directly and indirectly modulates prefrontal cortex (PFC) plasticity, which underpins higher-order cognitive functions. The prefrontal cortex (PFC) is responsible for advanced executive functions, working memory and cognitive control, and its normal function is highly dependent on neuroplasticity. The regulation of PFC plasticity by LT is mainly reflected in the following two aspects:

#### 4.2.1 Structural remodeling and molecular regulation

In the PFC, LT has been shown to modulate monoaminergic tone; animal studies indicate that light deprivation damages monoamine neurons, whereas normal lighting maintains and elevates serotonin, dopamine, and norepinephrine levels (75). These monoamines act as neuromodulators that directly regulate PFC neuronal excitability and synaptic transmission, providing the necessary neurochemical environment for cognitive function. The BDNF-TrkB pathway is similarly activated in the PFC, specifically engaging the high-affinity TrkB receptor to promote memory consolidation (76). Morphologically, chronic transcranial photobiomodulation increases dendritic spine density in the cerebral cortex, including prefrontal regions (77). Furthermore, LT appears to improve the local inflammatory milieu by lowering IL-6, NF-κB, and TNF-*α* levels and by restraining stress-induced astrocytic and microglial reactivity (71).

#### 4.2.2 Network functional plasticity

The cognitive function of the PFC needs to be realized through the formation of large-scale functional networks with other brain regions. LT can induce beneficial reorganization of these networks, which specifically includes:

Optimization of the Dorsolateral Prefrontal Cortex (dlPFC) Network: The dlPFC is the core brain region for cognitive control.

A preliminary study combining LT with neurofeedback training observed specific changes in the functional connection of the dlPFC: the functional connection between the dlPFC and the posterior cingulate cortex and dorsal striatum… is enhanced, while the connection between the dlPFC and the posterior cingulate cortex and dorsal striatum (involved in goal-directed behavior) is enhanced, while the connection between the dlPFC and the insula (involved in interoception and negative emotions) and the default mode network (active during resting state and rumination) is weakened (78). This connection mode change of “strengthening functional pathways-weakening interference pathways” is believed to be a key mechanism for improving task concentration and cognitive flexibility by reducing endogenous interference (such as rumination) and strengthening cognitive executive pathways. Given that LT promotes structural and molecular plasticity in both the PFC and hippocampus, and that light exposure is known to modulate widespread brain networks including prefrontal-hippocampal circuitry (79), it is plausible that LT concomitantly strengthens prefrontal-hippocampal functional coupling. However, direct imaging evidence in the context of LT remains limited and represents an important area for future investigation. It is plausible that LT concomitantly strengthens prefrontal-hippocampal functional coupling; however, direct imaging evidence in the context of LT remains limited and represents an important area for future investigation.

### 4.3 Neural oscillation entrainment: rapid regulation of brain network dynamics

In addition to relatively slow structural and molecular changes, light (especially light flickering at a specific frequency) can quickly and rhythmically regulate brain network activity through the mechanism of neural oscillation entrainment, which represents a form of functional and dynamic modulation of brain networks. Gamma oscillations (30–100 Hz) are hallmark rhythms of cognitive activities in a healthy brain, such as attention and memory binding, and are crucial for cognitive functions like memory and inter-regional brain communicatio (80, 81). In recent years, 40 Hz gamma light stimulation has attracted widespread attention as an emerging intervention method: this stimulation can “drive” the brain to produce synchronous gamma oscillations through a specific frequency. Studies have shown that this gamma entrainment can quickly improve attention, enhance the neural activity in the hippocampal region, and be directly related to the recovery of memory function (82–84); moreover, this rhythmic driving can also enhance the information processing efficiency of the neural network, providing a more comprehensive perspective for understanding the “rapid onset” mechanism of LT (85).

The non-image-forming (NIF) functions mediated by ipRGCs-circadian photoentrainment, light-inhibited melatonin secretion, and the pupillary light reflex (PLR)-are all engaged by LT. Notably, PLR sensitivity has been proposed as a biomarker of ipRGC functional integrity and may correlate with depression severity. Thus, it has been suggested that PLR assessment could provide an objective tool to evaluate the state of ipRGC pathways targeted by LT, potentially aiding in patient stratification and treatment monitoring (86), though further validation is required. PLR sensitivity has been proposed as a biomarker of ipRGC functional integrity and may correlate with depression severity (80), though further validation is required.

### 4.4 Multi-level plasticity mechanism framework: systematic summary and understanding of the relationship between related mechanisms

**Figure 3:**
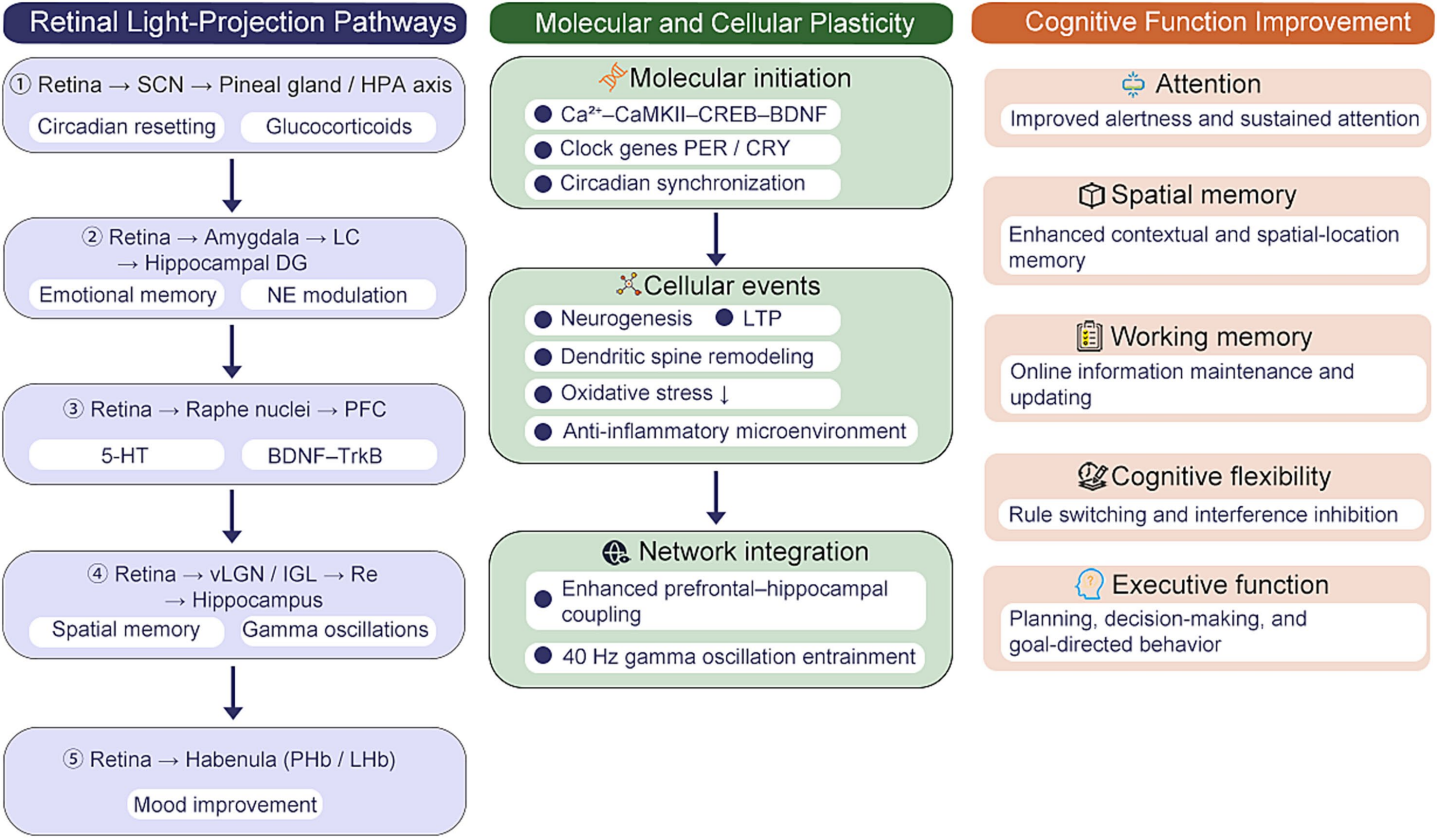
A multi-level integrative framework of light-induced neuroplasticity. Light signals are conveyed via four major retinofugal pathways (left panel). These pathways converge to trigger molecular initiation (Ca^2+^-CaMKII-CREB-BDNF axis, clock gene regulation), which leads to cellular plastic events (neurogenesis, synaptic strengthening, microenvironment improvement). Finally, network-level integration, including prefrontal-hippocampal coupling and gamma entrainment, translates these changes into improvements in specific cognitive domains (right panel).

To systematically explain how light converts external physical signals into internal structural changes in the brain, this study constructs a multi-level plasticity mechanism framework (Figure 3). This framework reveals that light achieves neural circuit remodeling and cognitive function improvement through three progressive levels of “molecular pathway initiation-cellular event execution-network function integration.” As summarized in Table 2, each retinofugal pathway converges on distinct but overlapping plasticity processes, collectively giving rise to improvements in multiple cognitive domains.

**Table 2:** Summary of retinofugal pathways, associated neuroplastic events, and cognitive domains.

Retinofugal pathway	Key brain regions	Neuroplastic events induced	Cognitive domains affected	References
Retina → SCN → Pineal/HPA axis	SCN, pineal gland, hippocampus	Circadian rhythm stabilization and sleep improvement; glucocorticoid rhythm normalization; PER1-dependent modulation of synaptic plasticity	Attention, memory consolidation, executive function	(34, 37, 41–44, 47, 48)
Retina → Amygdala (CeA) → LC → Hippocampal DG	Central amygdala, locus coeruleus, dentate gyrus	NE-mediated enhancement of spatial memory retrieval; amygdala-hippocampal *γ* oscillation coupling; synaptic strengthening	Spatial memory, emotional memory, fear extinction	(49–51, 53)
Retina → Raphe (DR) → PFC	Dorsal raphe nucleus, prefrontal cortex	5-HT-dependent modulation of prefrontal plasticity; BDNF–TrkB activation; dendritic spine remodeling	Working memory, cognitive flexibility, impulse control	(54–56, 59–61, 75, 76)
Retina → vLGN/IGL → Reuniens → Hippocampus	vLGN/IGL, nucleus reuniens, hippocampus	Enhanced hippocampal γ oscillations; promotion of neurogenesis; anti-inflammatory effects	Spatial memory, novelty recognition, learning	(35, 64, 65, 71, 72)
Retina → PHb/LHb (emotion-related)	Perihabenular nucleus, lateral habenula	Mood improvement via habenular modulation; indirect facilitation of cognitive recovery	Emotional regulation, motivation (indirectly supports cognition)	(61–63)

#### 4.4.1 Molecular initiation mechanism: activation of core signaling pathways

Accumulating evidence from preclinical models indicates that through neural projections such as the retinohypothalamic tract, light can indirectly or directly activate two core molecular signal axes in key brain regions, which is proposed to be the starting link of the plasticity process:

Ca^2+^-CaMKII-CREB-BDNF Signal Axis: Light triggers an increase in intracellular Ca^2+^ concentration, thereby activating CaMKII; activated CaMKII phosphorylates and activates the transcription factor CREB, which then enters the nucleus to initiate the transcription of the BDNF gene; after the synthesis and secretion of BDNF protein, it binds to the high-affinity receptor TrkB through autocrine or paracrine effects, activating downstream pro-survival and plasticity pathways such as MAPK/ERK and PI3K/Akt, and finally driving the synthesis of synaptic proteins (such as PSD-95, Synaptophysin), providing a molecular basis for structural plasticity.

Rhythm Gene Regulatory Axis: Light indirectly or directly synchronizes the local circadian rhythm of brain regions such as the hippocampus and prefrontal cortex by regulating the SCN core pacemaker. Its molecular core lies in regulating the rhythmic expression of nuclear clock genes (such as PER1, PER2, CRY): CRY and PER proteins form a complex through a transcription-translation feedback loop, which jointly inhibits the transcriptional activity of CLOCK-BMAL1; light may affect the hippocampal rhythm by regulating the stability or nuclear translocation of the CRY-PER complex (such as recruiting CK1δ) (87). The transcriptional activation of the PER1 gene is the core of light-regulated rhythm, and its expression level is directly related to the formation of long-term memory-the lack of PER1 can lead to impaired long-term memory, while light can optimize the memory formation process by targeting the regulation of hippocampal PER1 expression (67). It has been hypothesized that, through the transcription-translation feedback loop mechanism, these genes control the transcription of a large number of downstream clock-controlled genes, thereby contributing to the circadian time window that gates neuroplastic events (such as LTP, neurogenesis).

#### 4.4.2 Cellular plasticity events: functional execution of molecular signals

After the activation of the above molecular pathways, the following key plasticity events are specifically executed and manifested at the cellular level:

Enhanced Synaptic Plasticity: It is the most direct manifestation of plasticity. Driven by molecular signals, neurons undergo structural remodeling such as increased dendritic spine density and thickened postsynaptic dense substances, accompanied by the induction and maintenance of LTP, thereby stabilizing memory traces.

Hippocampal Neurogenesis: In the DG region of the hippocampus, neurotrophic signals such as BDNF promote the proliferation, differentiation of neural stem cells/progenitor cells and their maturation into new neurons; these new neurons are integrated into the existing neural network, potentially providing new computing units for the circuit, which is considered to be one cellular basis for the improvement of cognitive function.

Maintenance of Microenvironment Homeostasis: Through inhibiting inflammatory pathways such as NF-κB, light reduces the levels of pro-inflammatory factors (TNF-*α*, IL-6) and alleviates oxidative stress, creating an anti-inflammatory and anti-oxidative supportive microenvironment for the above fragile plasticity processes.

#### 4.4.3 System network integration: from cellular plasticity to cognitive output

The plastic changes of individual cells need to be finally integrated at the neural network level to achieve the overall improvement of cognitive function, which specifically includes:

Functional Coupling of the Prefrontal-Hippocampal Circuit: The neural circuit between the hippocampus and the PFC is strengthened through the above-mentioned plastic changes, and its functional connection is enhanced, directly supporting the improvement of cognitive flexibility and contextual memory.

Neural Oscillation Entrainment: As a relatively independent fast pathway, light stimulation at a specific frequency (such as 40 Hz) can directly drive the cerebral cortex to produce synchronous gamma oscillations, optimizing the efficiency of neural information processing. This transient plasticity at the network level complements the slow structural plasticity mediated by BDNF, jointly forming a complete effect of “rapid onset” and “long-term improvement” of LT.

### 4.5 Alternative and complementary mechanisms

Although our model emphasizes retinofugal-driven neuroplasticity, alternative and complementary mechanisms likely contribute to LT’s cognitive benefits. For instance, LT realigns circadian rest-activity cycles and improves sleep architecture (41, 42), changes that alone can enhance memory consolidation and prefrontal function. Systemic factors, including reduced peripheral pro-inflammatory cytokines (71), may also act in parallel to support brain health. Thus, the overall effect of LT should be viewed as arising from a network of interacting central and peripheral processes, with retinofugal pathways serving as a major input route. Systemic factors, including reduced peripheral pro-inflammatory cytokines (71), may also act in parallel to support brain health, but direct evidence linking LT to these systemic changes is still emerging.

## 5 Summary and outlook: the research path connecting “projection” and “plasticity”

The framework established here, bridging specific retinofugal projections with distinct forms of neuroplasticity, opens several promising avenues for future research. With “from retinal projections to neuroplasticity” as the central theme, this review synthesizes evidence to construct a comprehensive mechanistic framework of LT improving the cognitive function of depression: with ipRGCs as the only starting point, peripheral physical light signals are accurately transmitted to the distributed brain network through multiple neural projection pathways with different spatial distributions and specific functions (such as the retina-SCN rhythm pathway, the retina -limbic system fast pathway, the retina-thalamus direct pathway, etc.); after the convergence of these signals, by regulating key molecules such as neurotrophic factors, clock genes, neurotransmitters and inflammatory factors, multi-dimensional neuroplastic changes (including neurogenesis, synaptic remodeling, network reorganization) are induced in core cognitive brain regions such as the hippocampus and prefrontal cortex, and finally the consolidation and improvement of cognitive function are realized.

Looking forward to the future, in order to completely connect the complete path from “projection” to “plasticity” and transform the findings of basic research into clinical practice, the field needs to conduct in-depth exploration in the following directions:

### 5.1 Accurate localization and functional analysis at the projection level

Make full use of cutting-edge technologies such as optogenetics, chemogenetics and trans-synaptic virus tracing to accurately manipulate specific subtypes of ipRGCs or their specific downstream projection fibers in animal models, and verify the necessity and sufficiency of a certain pathway for specific plastic events (such as hippocampal neurogenesis vs. prefrontal dendritic spine remodeling) or cognitive behaviors (such as working memory vs. cognitive flexibility). This is a key link in constructing a detailed map of the LT mechanism.

### 5.2 Exploration of dynamic processes and biomarkers at the plasticity level

Study the dynamic impact of different light parameters (such as single acute irradiation vs. long-term chronic treatment) on the plasticity process; actively search for biomarkers that can reflect the state of neuroplasticity *in vivo* and non-invasively, such as through multi-modal magnetic resonance (measuring hippocampal volume, resting-state functional connection), electroencephalography (measuring event-related potentials, oscillation power in specific frequency bands) or the levels of BDNF and inflammatory factors in peripheral blood, to objectively evaluate and monitor the efficacy of LT. Furthermore, it will be important to validate PLR sensitivity as a clinically feasible biomarker for ipRGC function and to determine its utility in predicting and monitoring LT efficacy (80).

### 5.3 Promotion of clinical transformation and individualized precision light therapy

These mechanistic insights pave the way for biomarker-guided, individualized LT protocols. For example, assessing ipRGC function via PLR (80), determining circadian phase through dim-light melatonin onset, and profiling baseline cognitive deficits could enable tailored selection of wavelength, intensity, and timing, thereby maximizing therapeutic efficacy. Based on the comprehensive evaluation of the functional state of the patient’s specific projection pathway (such as evaluating the function of ipRGCs) through the pupillary light reflex (80), endogenous biological clock type, genetic background (such as BDNF, clock gene polymorphism) and baseline brain network characteristics, develop a truly individualized precision LT plan, including “tailor-made” optimal light wavelength, intensity, timing and treatment course for different patients. At the clinical research level, future personalized treatment trials could be designed around biomarkers, such as assigning different light parameters according to the patient’s pupillary reflex characteristics, melatonin secretion curve or fMRI baseline activity, to evaluate whether treatment accuracy can be improved. In addition, it is also necessary to expand the research perspective to the comorbid population (such as post-stroke depression, Alzheimer’s disease with depression) and special populations (such as perinatal patients), and systematically evaluate the benefits of LT in these complex clinical situations.

### 5.4 Exploration of synergistic therapy and new technology application

Systematically study the synergistic effect of LT with other existing treatment methods (such as antidepressant drugs, cognitive behavioral therapy, transcranial magnetic stimulation); at the same time, embrace the development of new technologies, such as constructing a closed-loop LT system-which can automatically and dynamically adjust light parameters according to real-time physiological signals (such as electroencephalography, heart rate variability) to achieve “on-demand treatment.” This represents a promising direction for future technological development, and has the potential to break through the limitations of current fixed-parameter treatment. In addition, intervention models based on new technologies such as wearable light therapy devices and transcranial photobiomodulation will further enrich the multi-level intervention system for cognitive impairment in depression.

By focusing on the central proposition of “from retinal projections to neuroplasticity,” this review not only provides a panoramic mechanism explanation from micro to macro and from the starting point to the end point for understanding LT, an “ancient yet novel” intervention method, but more importantly, lays a solid theoretical foundation for future circuit-targeted intervention strategies for cognitive impairment in psychiatric and neurological diseases, and points out the research directions with clinical transformation value.

## References

[1] Soto NN Gaspar P Bacci A. Not just a mood disorder─is depression a neurodevelopmental, cognitive disorder? Focus on prefronto-thalamic circuits. ACS Chem Neurosci. (2024) 15:1611–8. doi: 10.1021/acschemneuro.3c00828, 38580316 PMC11027097

[2] Mac Giollabhui N Alloy LB Hartman CA. Investigating whether depressed youth exhibiting elevated C reactive protein perform worse on measures of executive functioning, verbal fluency and episodic memory in a large, population based sample of Dutch adolescents. Brain Behav Immun. (2021) 94:369–80. doi: 10.1016/j.bbi.2020.08.030 32889083 PMC7921209

[3] Cariello AN Perrin PB Agudelo YR Olivera Plaza SL Quijano-Martínez MC Trujillo MA . Predictors of longitudinal depression trajectories after traumatic brain injury in Latin America: a multi-site study. NeuroRehabilitation. (2020) 46:205–12. doi: 10.3233/nre-192972 32083603

[4] Zainal NZ Kalita P Herr KJ. Cognitive dysfunction in Malaysian patients with major depressive disorder: a subgroup analysis of a multicountry, cross-sectional study. Asia Pac Psychiatry. (2019) 11:e12346. doi: 10.1111/appy.12346 30511420

[5] Diniz BS Sibille E Ding Y Tseng G Aizenstein HJ Lotrich F . Plasma biosignature and brain pathology related to persistent cognitive impairment in late-life depression. Mol Psychiatry. (2015) 20:594–601. doi: 10.1038/mp.2014.76 25092249 PMC4494754

[6] Armstrong NM Carlson MC Schrack J Xue QL Carnethon MR Rosano C . Late-life depressive symptoms as partial mediators in the associations between subclinical cardiovascular disease with onset of mild cognitive impairment and dementia. Am J Geriatr Psychiatry. (2018) 26:559–68. doi: 10.1016/j.jagp.2017.11.004 29254675 PMC5940555

[7] Glanz BI Kletenik I Singhal T Zurawski JD Chitnis T Weiner HL . Subjective cognitive function in individuals with multiple sclerosis: associations with objective cognitive function, anxiety, depression, and fatigue. J Neurol Sci. (2025) 475:123593. doi: 10.1016/j.jns.2025.123593 40580802

[8] Maruani J Geoffroy PA. Bright light as a personalized precision treatment of mood disorders. Front Psych. (2019) 10:85. doi: 10.3389/fpsyt.2019.00085 PMC6405415 30881318

[9] Tao L Jiang R Zhang K Qian Z Chen P Lv Y . Light therapy in non-seasonal depression: an update meta-analysis. Psychiatry Res. (2020) 291:113247. doi: 10.1016/j.psychres.2020.113247 32622169

[10] Li X Li X. The antidepressant effect of light therapy from retinal projections. Neurosci Bull. (2018) 34:359–68. doi: 10.1007/s12264-018-0210-1 29430586 PMC5856726

[11] Askalsky P Iosifescu DV. Transcranial photobiomodulation for the management of depression: current perspectives. Neuropsychiatr Dis Treat. (2019) 15:3255–72. doi: 10.2147/ndt.S188906, 31819453 PMC6878920

[12] Salehpour F Rasta SH. The potential of transcranial photobiomodulation therapy for treatment of major depressive disorder. Rev Neurosci. (2017) 28:441–53. doi: 10.1515/revneuro-2016-0087 28231069

[13] Iosifescu DV Norton RJ Tural U Mischoulon D Collins K McDonald E . Very low-level transcranial photobiomodulation for major depressive disorder: the ELATED-3 multicenter, randomized, sham-controlled trial. J Clin Psychiatry. (2022) 83:21m14226. doi: 10.4088/JCP.21m14226 35950904

[14] Hao W Dai X Wei M Li S Peng M Xue Q . Efficacy of transcranial photobiomodulation in the treatment for major depressive disorder: a TMS-EEG and pilot study. Photodermatol Photoimmunol Photomed. (2024) 40:e12957. doi: 10.1111/phpp.12957 38470033

[15] Chang CH Liu CY Chen SJ Tsai HC. Efficacy of light therapy on nonseasonal depression among elderly adults: a systematic review and meta-analysis. Neuropsychiatr Dis Treat. (2018) 14:3091–102. doi: 10.2147/ndt.S180321, 30532540 PMC6241691

[16] Seok JW Kim JD. Light therapy for older people with depressive symptoms: systematic review and meta-analysis. J Clin Med. (2024) 13:103498. doi: 10.3390/jcm13226982, 39598126 PMC11594984

[17] Tseng PT Chen YW Tu KY Chung W Wang HY Wu CK . Light therapy in the treatment of patients with bipolar depression: a meta-analytic study. Eur Neuropsychopharmacol. (2016) 26:1037–47. doi: 10.1016/j.euroneuro.2016.03.001, 26993616

[18] Zhou TH Dang WM Ma YT Hu CQ Wang N Zhang GY . Clinical efficacy, onset time and safety of bright light therapy in acute bipolar depression as an adjunctive therapy: a randomized controlled trial. J Affect Disord. (2018) 227:90–6. doi: 10.1016/j.jad.2017.09.038 29053981

[19] Chen G Guo Z Chen P Yang Z Yan H Sun S . Bright light therapy-induced improvements of mood, cognitive functions and cerebellar functional connectivity in subthreshold depression: a randomized controlled trial. Int J Clin Health Psychol. (2024) 24:100483. doi: 10.1016/j.ijchp.2024.100483, 39101053 PMC11296024

[20] Zhu G Tong Q Ye X Li J Zhou L Sun P . Phototherapy for cognitive function in patients with dementia: a systematic review and meta-analysis. Front Aging Neurosci. (2022) 14:936489. doi: 10.3389/fnagi.2022.936489, 35847661 PMC9284896

[21] Canazei M Dick M Pohl W Weninger J Hubel N Staggl S . Impact of repeated morning bright white light exposures on attention in a simulated office environment. Sci Rep. (2023) 13:8730. doi: 10.1038/s41598-023-35689-1, 37253767 PMC10229615

[22] Bjerrum LB Nordhus IH Sørensen L Wulff K Bjorvatn B Flo-Groeneboom E . Acute effects of light during daytime on central aspects of attention and affect: a systematic review. Biol Psychol. (2024) 192:108845. doi: 10.1016/j.biopsycho.2024.108845, 38981576

[23] Zou H Zhou H Yan R Yao Z Lu Q. Chronotype, circadian rhythm, and psychiatric disorders: recent evidence and potential mechanisms. Front Neurosci. (2022) 16:811771. doi: 10.3389/fnins.2022.811771, 36033630 PMC9399511

[24] Levitan RD Levitt AJ Michalak EE Morehouse R Ramasubbu R Yatham LN . Appetitive symptoms differentially predict treatment response to fluoxetine, light, and placebo in nonseasonal major depression. J Clin Psychiatry. (2018) 79:17m11856. doi: 10.4088/JCP.17m11856 30063303

[25] Onega LL Pierce TW Epperly L. Bright light therapy to treat depression in individuals with mild/moderate or severe dementia. Issues Ment Health Nurs. (2018) 39:370–3. doi: 10.1080/01612840.2018.1437648, 29509051

[26] Suzuki Y Nakauchi S Liao HI. Selective activation of ip RGC modulates working memory performance. PLoS One. (2025) 20:e0327349. doi: 10.1371/journal.pone.0327349, 40587501 PMC12208440

[27] Chen X Liu L Mei H Jiang Z Yan W Shi L . Efficacy evaluation and facial expressions biomarker of light therapy in youths with subthreshold depression: a randomized control trial study. J Affect Disord. (2025) 380:357–65. doi: 10.1016/j.jad.2025.03.123 40122251

[28] Wang L Mao L Huang Z Switzer JA Hess DC Zhang Q. Photobiomodulation: shining a light on depression. Theranostics. (2025) 15:362–83. doi: 10.7150/thno.104502, 39744683 PMC11671386

[29] Petrowski K Buehrer S Niedling M Schmalbach B. The effects of light exposure on the cortisol stress response in human males. Stress. (2021) 24:29–35. doi: 10.1080/10253890.2020.1741543 32160826

[30] Pan D Hu G Li J Wang Z Chen Y Cao J. Blue light damages retinal ganglion cells via endoplasmic reticulum stress and autophagy in chickens. Invest Ophthalmol Vis Sci. (2025) 66:3. doi: 10.1167/iovs.66.1.3, 39745679 PMC11702841

[31] Wong NA Bahmani H. A review of the current state of research on artificial blue light safety as it applies to digital devices. Heliyon. (2022) 8:e10282. doi: 10.1016/j.heliyon.2022.e10282, 36042717 PMC9420367

[32] Ashton A Foster RG Jagannath A. Photic entrainment of the circadian system. Int J Mol Sci. (2022) 23:729. doi: 10.3390/ijms23020729, 35054913 PMC8775994

[33] Mahoney HL Schmidt TM. The cognitive impact of light: illuminating ip RGC circuit mechanisms. Nat Rev Neurosci. (2024) 25:159–75. doi: 10.1038/s41583-023-00788-5 38279030

[34] Maruani J Geoffroy PA. Multi-level processes and retina-brain pathways of photic regulation of mood. J Clin Med. (2022) 11:448. doi: 10.3390/jcm11020448, 35054142 PMC8781294

[35] Huang X Huang P Huang L Hu Z Liu X Shen J . A visual circuit related to the nucleus reuniens for the spatial-memory-promoting effects of light treatment. Neuron. (2021) 109:347–362.e7. doi: 10.1016/j.neuron.2020.10.023 33171117

[36] Huang X Tao Q Ren C. A comprehensive overview of the neural mechanisms of light therapy. Neurosci Bull. (2024) 40:350–62. doi: 10.1007/s12264-023-01089-8, 37555919 PMC10912407

[37] Klett N Gompf HS Allen CN Cravetchi O Hablitz LM Gunesch AN . GABAergic signalling in the suprachiasmatic nucleus is required for coherent circadian rhythmicity. Eur J Neurosci. (2024) 60:6652–67. doi: 10.1111/ejn.16582, 39558544 PMC11612841

[38] Minami Y Yoshikawa T Nagano M Koinuma S Morimoto T Fujioka A . Transgenic rats expressing dominant negative BMAL1 showed circadian clock amplitude reduction and rapid recovery from jet lag. Eur J Neurosci. (2021) 53:1783–93. doi: 10.1111/ejn.15085 33351992

[39] Bhaskar R Narayanan KB Singh KK Han SS. Mapping the connection between circadian rhythms, metabolism, and neurodegeneration: exploring therapeutic strategies. Curr Alzheimer Res. (2025) 22:678–97. doi: 10.2174/0115672050381989250626071304 40676783

[40] Guo Q Qiao D Zhai P Zhang R Wen Y Liu P . The relationship between the circadian protein PER and adolescent depression: the mediating effect of aberrant functional connectivity of suprachiasmatic nucleus-orbitofrontal cortex. J Psychiatr Res. (2025) 190:225–34. doi: 10.1016/j.jpsychires.2025.08.001 40795660

[41] Dedmari T Ramzan S Masoodi MH Hassan Mir R. Detailed review of melatonin and its role in managing the symptoms of depression. Curr Mol Med. (2026) 26:8–32. doi: 10.2174/0115665240315801250127113448 39917910

[42] Li Z Shu Y Liu Q Liu D Xie S Wei M . Sleep deprivation activated AMPK/FOXO3a signaling mediates pineal autophagy impairment to reduce melatonin secretion in CUMS + SD rats leading to depression combined with insomnia. Neurosci Lett. (2025) 848:138091. doi: 10.1016/j.neulet.2024.138091 39710185

[43] Bering T Blancas-Velazquez AS Rath MF. Circadian clock genes are regulated by rhythmic corticosterone at physiological levels in the rat hippocampus. Neuroendocrinology. (2023) 113:1076–90. doi: 10.1159/000533151, 37517388 PMC10614510

[44] Rawashdeh O Parsons R Maronde E. Clocking in time to gate memory processes: the circadian clock is part of the ins and outs of memory. Neural Plast. (2018) 2018:6238989. doi: 10.1155/2018/6238989, 29849561 PMC5925033

[45] Tsuno Y Mieda M. Circadian rhythm mechanism in the suprachiasmatic nucleus and its relation to the olfactory system. Front Neural Circuits. (2024) 18:1385908. doi: 10.3389/fncir.2024.1385908, 38590628 PMC11000122

[46] Su X Tang Y Zhong Y Liu Y. Suprachiasmatic nucleus vasoactive intestinal peptide neurons mediate light-induced transient forgetting. Neurosci Bull. (2025) 41:2025–35. doi: 10.1007/s12264-025-01456-7, 40670769 PMC12569329

[47] Bu Z Li X Shi J Qin Q Zhang H Qiu Y . Ip RGCs sensitive blue light exposure promotes the robustness of circadian and neural stem cells in sleep deprived conditions. Stem Cells Int. (2025) 2025:8828183. doi: 10.1155/sci/8828183, 40746442 PMC12313382

[48] Campbell I Sharifpour R Balda Aizpurua JF Beckers E Paparella I Berger A . Regional response to light illuminance across the human hypothalamus. eLife. (2024) 13:RP96576. doi: 10.7554/eLife.96576, 39466317 PMC11517251

[49] Puig MV Gulledge AT. Serotonin and prefrontal cortex function: neurons, networks, and circuits. Mol Neurobiol. (2011) 44:449–64. doi: 10.1007/s12035-011-8214-0, 22076606 PMC3282112

[50] Fernandez DC Fogerson PM Lazzerini Ospri L Thomsen MB Layne RM Severin D . Light affects mood and learning through distinct retina-brain pathways. Cell. (2018) 175:71–84.e18. doi: 10.1016/j.cell.2018.08.004, 30173913 PMC6190605

[51] Shang M Shen M Xu R Du J Zhang J OuYang D . Moderate white light exposure enhanced spatial memory retrieval by activating a central amygdala-involved circuit in mice. Commun Biol. (2023) 6:414. doi: 10.1038/s42003-023-04765-7, 37059729 PMC10104844

[52] McGlashan EM Poudel GR Jamadar SD Phillips AJK Cain SW. Afraid of the dark: light acutely suppresses activity in the human amygdala. PLoS One. (2021) 16:e0252350. doi: 10.1371/journal.pone.0252350, 34133439 PMC8208532

[53] Alkozei A Dailey NS Bajaj S Vanuk JR Raikes AC Killgore WDS. Exposure to blue. Front Neurol. (2021) 12:625443. doi: 10.3389/fneur.2021.625443, 33841300 PMC8032953

[54] Chen P Chen G Tang G Yang Z Ma W Chen C . Effects of light therapy on amygdala connectivity and serotoninergic system in young adults with subthreshold depression. Dialogues Clin Neurosci. (2025) 27:184–200. doi: 10.1080/19585969.2025.2503367, 40451207 PMC12128131

[55] Zheng J Anderson KL Leal SL Shestyuk A Gulsen G Mnatsakanyan L . Amygdala-hippocampal dynamics during salient information processing. Nat Commun. (2017) 8:14413. doi: 10.1038/ncomms14413, 28176756 PMC5309795

[56] Vitkute K Borghese F Hut RA Havekes R Meerlo P. Shedding light on brain function and mood: a role for the retinoraphe pathway in regulating serotonin. Biol Psychiatry. (2026) 99:614–26. doi: 10.1016/j.biopsych.2025.06.029, 40633887

[57] Zhang T Huang L Zhang L Tan M Pu M Pickard GE . ON and OFF retinal ganglion cells differentially regulate serotonergic and GABAergic activity in the dorsal raphe nucleus. Sci Rep. (2016) 6:26060. doi: 10.1038/srep26060, 27181078 PMC4867631

[58] Huang L Yuan T Tan M Xi Y Hu Y Tao Q . A retinoraphe projection regulates serotonergic activity and looming-evoked defensive behaviour. Nat Commun. (2017) 8:14908. doi: 10.1038/ncomms14908, 28361990 PMC5381010

[59] Grandjean J Corcoba A Kahn MC Upton AL Deneris ES Seifritz E . A brain-wide functional map of the serotonergic responses to acute stress and fluoxetine. Nat Commun. (2019) 10:350. doi: 10.1038/s41467-018-08256-w, 30664643 PMC6341094

[60] Yu W Zhang R Zhang A Mei Y. Deciphering the functions of raphe-hippocampal serotonergic and glutamatergic circuits and their deficits in Alzheimer's disease. Int J Mol Sci. (2025) 26:1234. doi: 10.3390/ijms26031234, 39941002 PMC11818420

[61] Sargin D Jeoung HS Goodfellow NM Lambe EK. Serotonin regulation of the prefrontal cortex: cognitive relevance and the impact of developmental perturbation. ACS Chem Neurosci. (2019) 10:3078–93. doi: 10.1021/acschemneuro.9b00073, 31259523

[62] Young SN. How to increase serotonin in the human brain without drugs. J Psychiatry Neurosci. (2007) 32:394–9. 18043762 PMC2077351

[63] Huang L Xi Y Peng Y Yang Y Huang X Fu Y . A visual circuit related to habenula underlies the antidepressive effects of light therapy. Neuron. (2019) 102:128–142.e8. doi: 10.1016/j.neuron.2019.01.037 30795900

[64] Shang Q Zhang L Xiao B Yang J Sun J Gao X . Juvenile bright light exposure ameliorates adult behavioral abnormalities by enhancing neurogenesis in a N-methyl-D-aspartate receptor dysfunction mouse model relevant for cognitive impairment in schizophrenia. Behav Brain Res. (2024) 472:115157. doi: 10.1016/j.bbr.2024.115157, 39047873

[65] Scharfman H Goodman J Macleod A Phani S Antonelli C Croll S. Increased neurogenesis and the ectopic granule cells after intrahippocampal BDNF infusion in adult rats. Exp Neurol. (2005) 192:348–56. doi: 10.1016/j.expneurol.2004.11.016, 15755552

[66] Hirakawa H Terao T Hatano K Shirahama M Kugimiya T Kohno K . Increased volume of the left hippocampal dentate gyrus after 4 weeks of bright light exposure in patients with mood disorders: a randomized controlled study. Transl Psychiatry. (2023) 13:394. doi: 10.1038/s41398-023-02688-9, 38102115 PMC10724173

[67] Kwapis JL Alaghband Y Kramár EA López AJ Vogel Ciernia A White AO . Epigenetic regulation of the circadian gene per 1 contributes to age-related changes in hippocampal memory. Nat Commun. (2018) 9:3323. doi: 10.1038/s41467-018-05868-0, 30127461 PMC6102273

[68] Soler JE Robison AJ Núñez AA Yan L. Light modulates hippocampal function and spatial learning in a diurnal rodent species: a study using male nile grass rat (*Arvicanthis niloticus*). Hippocampus. (2018) 28:189–200. doi: 10.1002/hipo.22822, 29251803 PMC5820160

[69] Shang M Zhang J Shen M Sun Z Gao P Li J . Bright light exposure induces dynamic changes of spatial memory in nocturnal rodents. Brain Res Bull. (2021) 174:389–99. doi: 10.1016/j.brainresbull.2021.06.019 34197939

[70] Jasińska M Jasek-Gajda E Ziaja M Litwin JA Lis GJ Pyza E. Light-modulated circadian synaptic plasticity in the somatosensory cortex: link to locomotor activity. Int J Mol Sci. (2024) 25:12870. doi: 10.3390/ijms252312870, 39684579 PMC11641775

[71] Salehpour F Mahmoudi J Kamari F Sadigh-Eteghad S Rasta SH Hamblin MR. Brain photobiomodulation therapy: a narrative review. Mol Neurobiol. (2018) 55:6601–36. doi: 10.1007/s12035-017-0852-4, 29327206 PMC6041198

[72] Eshaghi E Sadigh-Eteghad S Mohaddes G Rasta SH. Transcranial photobiomodulation prevents anxiety and depression via changing serotonin and nitric oxide levels in brain of depression model mice: a study of three different doses of 810 nm laser. Lasers Surg Med. (2019) 51:634–42. doi: 10.1002/lsm.23082 30883832

[73] Feng Z Li Q He Z Yu B Mi T Niu J . Hypothalamic orexin projections to the hippocampal CA1 region alleviate cognitive and synaptic plasticity impairments induced by blue light exposure. CNS Neurosci Ther. (2025) 31:e70551. doi: 10.1111/cns.70551, 40820617 PMC12358687

[74] Berbegal-Sáez P Gallego-Landin I Macía J Valverde O. Irregular light schedules disrupt daily rhythms and dysregulate genes involved in neuroplasticity, motivation, and stress responses. Pharmacol Biochem Behav. (2025) 255:174075. doi: 10.1016/j.pbb.2025.174075 40749778

[75] Gonzalez MM Aston-Jones G. Light deprivation damages monoamine neurons and produces a depressive behavioral phenotype in rats. Proc Natl Acad Sci USA. (2008) 105:4898–903. doi: 10.1073/pnas.0703615105, 18347342 PMC2290795

[76] Sun W Yang Y Chen X Cheng Y Li X An L. Light promotes neural correlates of fear memory via enhancing brain-derived neurotrophic factor (BDNF) expression in the prelimbic cortex. ACS Chem Neurosci. (2021) 12:1802–10. doi: 10.1021/acschemneuro.1c00081 33961393

[77] Cardoso FDS Serra FT Coimbra NC Gonzalez-Lima F Gomes da Silva S. Transcranial photobiomodulation changes neuronal morphology in the cerebral cortex of rats. Neurosci Lett. (2022) 781:136681. doi: 10.1016/j.neulet.2022.136681 35569700

[78] Vandewalle G Maquet P Dijk DJ. Light as a modulator of cognitive brain function. Trends Cogn Sci. (2009) 13:429–38. doi: 10.1016/j.tics.2009.07.004 19748817

[79] Godet A Serrand Y Léger B Moirand R Bannier E Val-Laillet D . Functional near-infrared spectroscopy-based neurofeedback training targeting the dorsolateral prefrontal cortex induces changes in cortico-striatal functional connectivity. Sci Rep. (2024) 14:20025. doi: 10.1038/s41598-024-69863-w, 39198481 PMC11358514

[80] Maruani J Vissouze L Hebert M Rach H Zehani F Lejoyeux M . Pupillary response to blue light as a biomarker of seasonal pattern in major depressive episode: a clinical study using pupillometry. Psychiatry Res. (2025) 344:116333. doi: 10.1016/j.psychres.2024.116333 39721100

[81] Traikapi A Konstantinou N. Gamma oscillations in Alzheimer's disease and their potential therapeutic role. Front Syst Neurosci. (2021) 15:782399. doi: 10.3389/fnsys.2021.782399, 34966263 PMC8710538

[82] Manippa V Filardi M Vilella D Logroscino G Rivolta D. Gamma (60 Hz) auditory stimulation improves intrusions but not recall and working memory in healthy adults. Behav Brain Res. (2024) 456:114703. doi: 10.1016/j.bbr.2023.114703, 37806563

[83] Sahin L Figueiro MG. Flickering red-light stimulus for promoting coherent 40Hz neural oscillation: a feasibility study. J Alzheimer's Dis. (2020) 75:911–21. doi: 10.3233/jad-200179, 32390635 PMC8083946

[84] Lin Z Hou G Yao Y Zhou Z Zhu F Liu L . 40-Hz blue light changes hippocampal activation and functional connectivity underlying recognition memory. Front Hum Neurosci. (2021) 15:739333. doi: 10.3389/fnhum.2021.739333, 34975431 PMC8716555

[85] Ferreira AC Castellano JM. Leaving the lights on using gamma entrainment to protect against neurodegeneration. Neuron. (2019) 102:901–2. doi: 10.1016/j.neuron.2019.05.020 31170394

[86] Petro NM Webert LK Springer SD Okelberry HJ John JA Horne LK . Optimal gamma-band entrainment of visual cortex. Hum Brain Mapp. (2024) 45:e26775. doi: 10.1002/hbm.26775, 38970249 PMC11226544

[87] Cao X Yang Y Selby CP Liu Z Sancar A. Molecular mechanism of the repressive phase of the mammalian circadian clock. Proc Natl Acad Sci USA. (2021) 118:e2021174118. doi: 10.1073/pnas.2021174118, 33443219 PMC7812753

[88] Strong RE Marchant BK Reimherr FW Williams E Soni P Mestas R. Narrow-band blue-light treatment of seasonal affective disorder in adults and the influence of additional nonseasonal symptoms. Depress Anxiety. (2009) 26:273–8. doi: 10.1002/da.20538, 19016463

[89] Liu L Wu Z Lu Y Lu W Su G Zhou Z. Effects of phototherapy on biopterin, neopterin, tryptophan, and behavioral neuroinflammatory reaction in patients with post-stroke depression. Sci Rep. (2024) 14:18368. doi: 10.1038/s41598-024-68799-5, 39112627 PMC11306333

[90] Chojnacka M Antosik-Wójcińska AZ Dominiak M Bzinkowska D Borzym A Sokół-Szawłowska M . A sham-controlled randomized trial of adjunctive light therapy for non-seasonal depression. J Affect Disord. (2016) 203:1–8. doi: 10.1016/j.jad.2016.05.062 27267951

[91] Güzel Özdemir P Boysan M Smolensky MH Selvi Y Aydin A Yilmaz E. Comparison of venlafaxine alone versus venlafaxine plus bright light therapy combination for severe major depressive disorder. J Clin Psychiatry. (2015) 76:e645–54. doi: 10.4088/JCP.14m09376, 26035199

[92] Noda Y Taniguchi K Takano M Mimura Y Yanagisawa N Hayano M . Violet light photobiomodulation therapy for depression: a double-blind randomized crossover trial. J Affect Disord. (2025) 376:325–32. doi: 10.1016/j.jad.2025.02.031, 39961441

